# Supplementary material for: Multi‐dataset identification of innovative feature genes and molecular mechanisms in keratoconus
Source: J Cell Mol Med. 2024 Sep 19;28(18):e70079. doi: 10.1111/jcmm.70079 (PMC11412914; doi:10.1111/jcmm.70079)
Supplement: Supplementary file 2 — Material S2. [file JCMM-28-e70079-s002.zip › 7.GSEA analysis/1.ARL11/KEGG_DRUG_METABOLISM_CYTOCHROME_P450.html]

Details for gene set KEGG\_DRUG\_METABOLISM\_CYTOCHROME\_P450[GSEA]

|  || Dataset | ARL11.group.cls#h\_versus\_l |
| Phenotype | group.cls#h\_versus\_l |
| Upregulated in class | h |
| GeneSet | KEGG\_DRUG\_METABOLISM\_CYTOCHROME\_P450 |
| Enrichment Score (ES) | 0.27311668 |
| Normalized Enrichment Score (NES) | 0.8877397 |
| Nominal p-value | 0.6770833 |
| FDR q-value | 1.0 |
| FWER p-Value | 1.0 |
Table: GSEA Results Summary

  

Fig 1: Enrichment plot: KEGG\_DRUG\_METABOLISM\_CYTOCHROME\_P450      
 Profile of the Running ES Score & Positions of GeneSet Members on the Rank Ordered List

  

| SYMBOL | TITLE | RANK IN GENE LIST | RANK METRIC SCORE | RUNNING ES | CORE ENRICHMENT || 1 | MAOB | na | 1064 | 0.382 | 0.0166 | Yes |
| 2 | UGT2B7 | na | 1178 | 0.368 | 0.0589 | Yes |
| 3 | FMO5 | na | 1646 | 0.324 | 0.0855 | Yes |
| 4 | GSTA3 | na | 2651 | 0.258 | 0.0884 | Yes |
| 5 | UGT2B15 | na | 2914 | 0.244 | 0.1111 | Yes |
| 6 | UGT2B11 | na | 2923 | 0.244 | 0.1411 | Yes |
| 7 | CYP3A7 | na | 3477 | 0.220 | 0.1523 | Yes |
| 8 | UGT2B4 | na | 3576 | 0.216 | 0.1763 | Yes |
| 9 | ADH4 | na | 3617 | 0.215 | 0.2017 | Yes |
| 10 | UGT1A8 | na | 3762 | 0.209 | 0.2234 | Yes |
| 11 | CYP2C19 | na | 4519 | 0.182 | 0.2241 | Yes |
| 12 | UGT1A6 | na | 4803 | 0.172 | 0.2372 | Yes |
| 13 | GSTA2 | na | 5278 | 0.158 | 0.2431 | Yes |
| 14 | UGT2B28 | na | 5683 | 0.146 | 0.2494 | Yes |
| 15 | CYP1A2 | na | 5834 | 0.141 | 0.2626 | Yes |
| 16 | ADH6 | na | 6053 | 0.136 | 0.2731 | Yes |
| 17 | CYP2D6 | na | 7332 | 0.105 | 0.2492 | No |
| 18 | CYP3A5 | na | 7636 | 0.099 | 0.2527 | No |
| 19 | CYP2E1 | na | 8693 | 0.079 | 0.2321 | No |
| 20 | AOX1 | na | 8738 | 0.079 | 0.2406 | No |
| 21 | UGT2B10 | na | 9893 | 0.062 | 0.2149 | No |
| 22 | MGST2 | na | 10508 | 0.054 | 0.2038 | No |
| 23 | GSTT2 | na | 11478 | 0.042 | 0.1810 | No |
| 24 | ALDH3B1 | na | 11840 | 0.038 | 0.1753 | No |
| 25 | MAOA | na | 12281 | 0.034 | 0.1668 | No |
| 26 | MGST3 | na | 12526 | 0.031 | 0.1636 | No |
| 27 | CYP2B6 | na | 12739 | 0.029 | 0.1611 | No |
| 28 | GSTT1 | na | 13017 | 0.027 | 0.1564 | No |
| 29 | UGT2A3 | na | 13634 | 0.022 | 0.1413 | No |
| 30 | ALDH3B2 | na | 13954 | 0.019 | 0.1345 | No |
| 31 | CYP3A4 | na | 14527 | 0.015 | 0.1198 | No |
| 32 | ADH1B | na | 16426 | 0.001 | 0.0652 | No |
| 33 | GSTP1 | na | 17007 | -0.003 | 0.0489 | No |
| 34 | GSTO1 | na | 17080 | -0.004 | 0.0472 | No |
| 35 | GSTO2 | na | 17085 | -0.004 | 0.0476 | No |
| 36 | CYP2C9 | na | 17344 | -0.006 | 0.0408 | No |
| 37 | GSTK1 | na | 18167 | -0.012 | 0.0186 | No |
| 38 | ADH7 | na | 18569 | -0.014 | 0.0087 | No |
| 39 | CYP2C8 | na | 18873 | -0.016 | 0.0020 | No |
| 40 | GSTZ1 | na | 19033 | -0.017 | -0.0005 | No |
| 41 | GSTM5 | na | 20064 | -0.024 | -0.0272 | No |
| 42 | ALDH3A1 | na | 20262 | -0.026 | -0.0297 | No |
| 43 | CYP2C18 | na | 20676 | -0.029 | -0.0381 | No |
| 44 | UGT2A1 | na | 20689 | -0.029 | -0.0348 | No |
| 45 | CYP2A7 | na | 21232 | -0.033 | -0.0464 | No |
| 46 | GSTM3 | na | 21920 | -0.039 | -0.0614 | No |
| 47 | GSTM2 | na | 23101 | -0.051 | -0.0891 | No |
| 48 | ADH5 | na | 23842 | -0.060 | -0.1030 | No |
| 49 | CYP2A13 | na | 25078 | -0.077 | -0.1291 | No |
| 50 | GSTA4 | na | 25863 | -0.088 | -0.1408 | No |
| 51 | FMO4 | na | 25931 | -0.089 | -0.1317 | No |
| 52 | ALDH1A3 | na | 28596 | -0.146 | -0.1905 | No |
| 53 | GSTM1 | na | 28996 | -0.156 | -0.1827 | No |
| 54 | FMO1 | na | 29815 | -0.182 | -0.1837 | No |
| 55 | MGST1 | na | 31885 | -0.256 | -0.2118 | No |
| 56 | CYP3A43 | na | 32273 | -0.273 | -0.1891 | No |
| 57 | GSTM4 | na | 32561 | -0.292 | -0.1613 | No |
| 58 | ADH1C | na | 33242 | -0.340 | -0.1389 | No |
| 59 | FMO3 | na | 33332 | -0.347 | -0.0986 | No |
| 60 | ADH1A | na | 33507 | -0.360 | -0.0590 | No |
| 61 | GSTA5 | na | 33671 | -0.375 | -0.0173 | No |
| 62 | FMO2 | na | 33774 | -0.386 | 0.0275 | No |
Table: GSEA details [plain text format]

  

Fig 2: KEGG\_DRUG\_METABOLISM\_CYTOCHROME\_P450      
 Blue-Pink O' Gram in the Space of the Analyzed GeneSet

  

Fig 3: KEGG\_DRUG\_METABOLISM\_CYTOCHROME\_P450: Random ES distribution      
 Gene set null distribution of ES for **KEGG\_DRUG\_METABOLISM\_CYTOCHROME\_P450**
